# Triggers and mediators of epigenetic remodeling in plants

**DOI:** 10.3389/freae.2023.1188733

**Published:** 2023-05-23

**Authors:** Luca Comai

**Affiliations:** Department of Plant Biology and Genome Center, University of California, Davis, CA, United States

**Keywords:** chromatin remodeling, stress, epiallele, transposon, methylation, DNA, evolution

## Abstract

Plant epigenetic studies have revealed that developmental or environmental events can trigger both local and global epigenetic remodeling. In multiple cases, transposable elements (TE) respond to the trigger and act as mediators. Epigenetic remodeling results in mitotically and even meiotically persistent states that impact phenotype and could contribute to its plasticity. The challenge is to understand the mechanisms that trigger and mediate remodeling, their evolutionary role, and their potential in breeding.

## Introduction

Plants life cycles display great diversity, from the short sexual cycles of annuals such as arabidopsis and cultivated rice, to the clonal persistence of invasive weeds such as knotweed (Hollingsworth and Bailey, 2000) and cordgrass (Ainouche et al, 2009), and to the long lives of forest species, such as juniper, fir, and aspen (de Witte and Stöcklin, 2010). Plants are sessile and must adapt to their varying environment. Mitotically stable changes alone have great importance for large and long-lived clones that encounter great spatial and temporal environmental variation. For plants with shorter cycles, both mitotic and meiotically persistent epigenetic changes could play a role in fitness. The existence of this potential is a critical question at a time of accelerated climate change.

## Developmental epigenetics in plants

We have made progress in understanding programmed epigenetic switches occurring during reproductive development. These are recurring changes associated with the development of flowers, fruits and seeds. Flowering in arabidopsis is one of the best characterized epigenetic switches. FLOWERING LOCUS C (FLC), a repressor of flowering, integrates signals from three pathways, including one involving antisense RNAs, to undergo persistent silencing by the Polycomb Repressive Complex (PRC) and generational resetting by histone demethylases (Whittaker and Dean, 2017). Certain specialized tissues essential for reproduction undergo large-scale epigenetic changes: fleshy tissues in fruits, the central cell and endosperm, and the vegetative cell of pollen. These tissues are distinct, non-proliferating branches of plant development. Fleshy fruits exhibit two physiological ripening responses: climacteric ones, such as tomato, exhibit a postharvest, ethylene-triggered respiratory burst (Chalmers and Rowan, 1971; Gallusci et al, 2016). Non-climacteric, such as oranges, do not. In both types, ripening can be regulated by large-scale changes in DNA methylation (Gallusci et al, 2016). In tomato fruit, DNA methylation at many loci opposes the action of ethylene, preventing early ripening. Demethylating enzymes (Liu et al, 2015) remove methyl-C marks. Demethylation plays a role in strawberry, a non-climacteric fruit, and is mediated by downregulation of RNA-dependent DNA methylation (RdDM) (Cheng et al, 2018). The opposite happens in oranges, where non-climacteric ripening correlates with hypermethylation (Huang et al, 2019). In developing seed endosperm, a nutritional organ resulting from independent fertilization, parental imprinting of key seed factors depends on the maternal action of DNA demethylases and on the maternal establishment of PRC-dependent repressive chromatin (Gehring, 2013; Moreno-Romero et al, 2019). In the pollen vegetative cell, a non-dividing cell supporting male reproduction, epigenetic marks in transposable elements (TE) undergo programmed relaxation (Slotkin et al, 2009) and produce small RNA capable of interacting with sperm targets (Calarco and Martienssen, 2011). Small RNAs are thought to orchestrate epigenetic genomic changes at various stages of reproduction, including the haploid gametophytic stage although the specific mechanisms are still being elucidated (Martinez and Köhler, 2017). TEs are not the only genomic feature to lose a genomic mark persistent in the Soma: centromeres undergo programmed loss of their characteristic chromatin via proteasomal processing of sumoylated CENH3 (Mérai et al, 2014). Taken together, the epigenetic changes documented at the different developmental stages of flowering, fruiting, and seed production demonstrate the flexible and varied regulation of developmental chromatin marks and indicate a key role in reproductive fitness.

## Epigenetic responses to stress

Many studies have documented the effect of stress, biotic and abiotic, on epigenetic regulation: see Gallusci (2023) for a recent review. How does stress affect epigenetic regulation? A take-home lesson from FLOWERING LOCUS C regulation is that cold and day length signaling are integrated into the developmental regulation leading to epigenetic imprinting (Whittaker and Dean, 2017). Because many plant species are exposed to cold winters, they have evolved sophisticated chromatin-based mechanisms to ensure that they flower only in favorable times. Other environmental stresses recur. In most environments, even mesic ones, drought and heat periods are expected on a yearly basis. Multiple papers have documented environmentally triggered epigenetic changes affecting DNA and histone marks of key genes (Chang et al, 2020; Gallusci et al, 2023). These findings suggest that stress signals engender fitness-promoting epigenetic memories. It remains unclear what molecular mechanisms transduce environmental and physiological clues into epigenetic remodeling, what regulates their persistence, and to what degree these states endure through generations.

## Transposon regulation

Studies of epigenetic changes caused by tissue culture suggest that triggers are mediated by transposon regulation, consistent with early insights by Barbara (McClintock, 1984). Tissue culture involving shoot regeneration from organ explants or from single cell protoplasts combines multiple stresses because plant cells are exposed to wounding, to cell wall digestion by fungal enzymes (in the case of protoplasts), to unusual light and hormonal conditions, and are induced to proliferate and organize into shoot meristems. A few hours after exposure to protoplasting treatments, multiple species display strong activation of silent TEs (Pouteau et al, 1991; Hirochika, 1993; Pouteau et al, 1994). Rapid proliferation induced during tissue culture can destabilize DNA methylation (Borges et al, 2021), but the timing of TE induction suggests that at the least the early response is independent of the replication-dependent dilution of methyl-C observed during cell cycle acceleration. While methylation marks can be re-established, transposon activation has persistent effects. A disrupting effect depends on transposon excision and insertion at novel sites, resulting in DNA breaks, genome instability, and relocation of potential regulatory elements (Bennetzen and Wang, 2014). Indeed, both genetic changes from DNA instability and increased epigenetic changes contribute to somaclonal variation (Phillips et al, 1994; Kaeppler et al, 2000; Ong-Abdullah et al, 2015; Fossi et al, 2019; Borges et al, 2021). Another effect involves remodeling of chromatin states, particularly those associated with homologous copies of the activated transposons (Figure 1). Transposons can confer upon associated genes novel regulatory properties that depend on the TE epigenetic state (Bennetzen and Wang, 2014). Tissue culture of oil palm is associated with demethylation of the Karma TE and expression of the connected *MANTLED* gene resulting in undesirable mantling of the seed (Ong-Abdullah et al, 2015). Remarkably, transposons are often sensitive to stress and can confer stress sensitive regulation to nearby genes (Wessler, 1996; Cavrak et al, 2014). Independent insertion of two retroelements near the Ruby gene of sweet orange conferred cold-dependent activation of anthocyanin expression (Butelli et al, 2012). In rice, novel transposition of TE mPing to new sites resulted in stress-dependent activation of the connected genes (Naito et al, 2009). In summary, when a transposon or a transposon fragment is located near a gene, it can sensitize that gene to the status of cognate TEs. Because these are often sensitive to stress, the gene can become stress responsive. In conclusion, TE can sense multiple stresses and remodel the epigenome.

**FIGURE 1:**
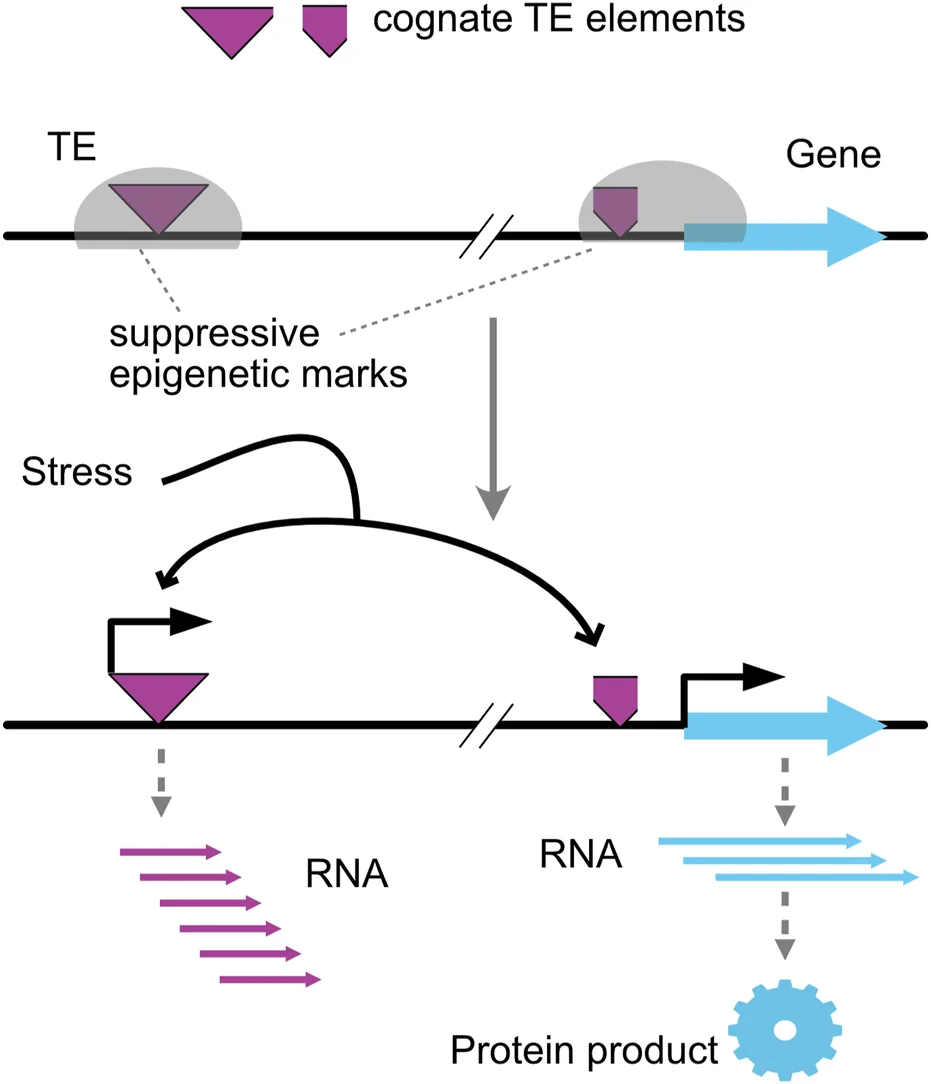
TE-mediated regulation of genes. A transposable element (TE) and its cognate element share epigenetic states. Stress can trigger the activation of the TE and induce expression of the proximal gene (Wessler, 1996; Butelli et al, 2012; Cavrak et al, 2014).

## Epialleles

An epiallele affects a trait without underlying DNA changes. Plant epialleles can confer qualitative and quantitative phenotypes and can persist through generations (Cubas et al, 1999; Mittelsten Scheid et al, 2003; Manning et al, 2006; Weigel and Colot, 2012; Quadrana et al, 2014; Zeng and Cheng, 2014; Baduel and Colot, 2021). Most persistent epialleles result from DNA methylation marks that reside inside a TE or spread from a TE. If a novel gene-TE association that results in altered regulation increases fitness, the polymorphism should experience positive selection. When associated with a persistent or potential epigenetic mark, sensitized loci can be identified when DNA methylation homeostasis is perturbed. For example, mutation in *MET1* or *DDM1*, respectively the major CpG methyltransferase and a chromatin factor needed for methylation maintenance, generates epialleles either by loss of methylation (Baduel and Colot, 2021), or by hypermethylation (Saze and Kakutani, 2007; Yi and Richards, 2008). Genome wide removal of methylation marks is largely reversible by complementation of the methylation defect, but some loci remain unmethylated providing allelic variation that can underlie QTL. Reversibility is affected by RdDM and the strength of the silencing signal provided by the cognate TE family (Baduel and Colot, 2021). Thus, biotic and abiotic stresses activate TEs, which in turn create and modulate epialleles. Expressed or silenced epialleles can flip state as a result of extreme life history events.

## Discussion

The effects of epigenetic marks on genes raises the questions of what initiates, destabilizes, and resets them. At least in part, the answers may lay in the co-evolution of parasitic genomic elements (and related viruses) with the host. As exemplified by the sophisticated sensing of the host state by lysogenic *λ* bacteriophage, selfish elements co-wire their regulation with host stress pathways (Rokney et al, 2008). In the case of plants, hormonal networks influence many regulatory decisions, both developmental and stress-dependent (Waadt et al, 2022): for example, imprinting-dependent phenotypes in developing seeds are exquisitely sensitive to regulation by flavonoids and auxins (Dilkes et al, 2008; Köhler et al, 2021). Epigenetic remodelers may thus directly or indirectly respond to hormonal regulation (Liu et al, 2015). Elucidating the changes of epigenetic states during development is a grand challenge. To what degree do different types of marks, such as those determined by the PRC, depend on TEs? Why do many TEs respond to stress? How do hormones participate in their regulation? Activation when the host is in danger makes good sense for viruses, but what do cell-bound elements gain from this strategy? How is cellular and developmental homeostasis coupled to epigenetic states?

Answering these questions will contribute to our basic understanding of epigenetic phenomena. Further, it will help elucidate the contribution of epigenetic state to evolution and the potential exploitation of epigenetic regulation by plant breeders. Over a quarter century ago, Rasmusson and Phillips proposed that unexpected phenotypic variation observed in relatively narrowly based breeding programs could, at least in part, be based on epigenetic changes (Rasmusson and Phillips, 1997). Methylation-dependent epialleles could thus contribute to variation useful for breeding (Tsaftaris and Polidoros, 1999). Experimental evidence supporting the influence of epialleles on quantitative traits relevant to agriculture has multiplied (Baduel and Colot, 2021). Mutation of mitochondrial MutS Homolog 1 affects DNA methylation homeostasis in the nuclear genome, influencing agronomic traits (Yang et al, 2015). It is possible, therefore, that natural and induced epigenetic manipulation could increase breeding efficiency (Springer and Schmitz, 2017; Gahlaut et al, 2020). To play an economic role, however, agronomic epialleles must be sufficiently stable, emphasizing the importance of understanding triggering and resetting mechanisms.

Notwithstanding the considerable interest in their potential, there is no conclusive evidence that epialleles have contributed to evolution and that they can be exploited for plant breeding. Skeptical views are useful: selection on homozygous lines that show phenotypic variation has failed (Charlesworth et al, 2017). Virtually all significant traits characterized have a genetic origin (Doebley et al, 2006; Weigel and Colot, 2012). Furthermore, most epialleles are genes sensitized by a genetic change, such as a TE insertion nearby or the formation of a transactive inverted repeat (Jablonka and Lamb, 1998; Melquist et al, 1999). These considerations should stimulate careful reflections on their role and on the type of evidence necessary to attribute an evolutionary role and to deploy them for breeding. For example, epialleles could serve as crutches conferring short term fitness in dire circumstances and facilitating the development of more persistent genetic solutions (Jablonka and Lamb, 1998; Kalisz and Purugganan, 2004). Plants are useful experimental systems to test these hypotheses.

In conclusion, scientific studies that describe the causes, mechanisms, and impact of changes in epigenetic states will broadly benefit the field of epigenetics and genome regulation.

## References

[B1] Ainouche M. L. Fortune P. M. Salmon A. Parisod C. Grandbastien M.-A. Fukunaga K. (2009). Hybridization, polyploidy and invasion: Lessons from spartina (poaceae). Biol. Invasions 11, 1159–1173. 10.1007/s10530-008-9383-2

[B2] Baduel P. Colot V. (2021). The epiallelic potential of transposable elements and its evolutionary significance in plants. Philosophical Trans. R. Soc. B Biol. Sci. 376, 20200123. 10.1098/rstb.2020.0123 PMC8059525 33866816

[B3] Bennetzen J. L. Wang H. (2014). The contributions of transposable elements to the structure, function, and evolution of plant genomes. Annu. Rev. Plant Biol. 65, 505–530. 10.1146/annurev-arplant-050213-035811 24579996

[B4] Borges F. Donoghue M. T. A. LeBlanc C. Wear E. E. Tanurdžić M. Berube B. (2021). Loss of small-RNA-directed DNA methylation in the plant cell cycle promotes germline reprogramming and somaclonal variation. Curr. Biol. 31, 591–600.e4. 10.1016/j.cub.2020.10.098 PMC9019819 33275892

[B5] Butelli E. Licciardello C. Zhang Y. Liu J. Mackay S. Bailey P. (2012). Retrotransposons control fruit-specific, cold-dependent accumulation of anthocyanins in blood oranges. Plant Cell 24, 1242–1255. 10.1105/tpc.111.095232 PMC3336134 22427337

[B6] Calarco J. P. Martienssen R. A. (2011). Genome reprogramming and small interfering RNA in the Arabidopsis germline. Curr. Opin. Genet. Dev. 21, 134–139. 10.1016/j.gde.2011.01.014 PMC3073301 21330131

[B7] Cavrak V. V. Lettner N. Jamge S. Kosarewicz A. Bayer L. M. Mittelsten Scheid O. (2014). How a retrotransposon exploits the plant’s heat stress response for its activation. PLoS Genet. 10, e1004115. 10.1371/journal.pgen.1004115 PMC3907296 24497839

[B8] Chalmers D. J. Rowan K. S. (1971). The climacteric in ripening tomato fruit. Plant Physiol. 48, 235–240. 10.1104/pp.48.3.235 PMC396839 16657771

[B9] Chang Y.-N. Zhu C. Jiang J. Zhang H. Zhu J.-K. Duan C.-G. (2020). Epigenetic regulation in plant abiotic stress responses. J. Integr. Plant Biol. 62, 563–580. 10.1111/jipb.12901 31872527

[B10] Charlesworth D. Barton N. H. Charlesworth B. (2017). The sources of adaptive variation. Proc. R. Soc. B Biol. Sci. 284, 20162864. 10.1098/rspb.2016.2864 PMC5454256 28566483

[B11] Cheng J. Niu Q. Zhang B. Chen K. Yang R. Zhu J.-K. (2018). Downregulation of RdDM during strawberry fruit ripening. Genome Biol. 19, 212. 10.1186/s13059-018-1587-x PMC6280534 30514401

[B12] Cubas P. Vincent C. Coen E. (1999). An epigenetic mutation responsible for natural variation in floral symmetry. Nature 401, 157–161. 10.1038/43657 10490023

[B13] de Witte L. C. Stöcklin J. (2010). Longevity of clonal plants: Why it matters and how to measure it. Ann. Bot. 106, 859–870. 10.1093/aob/mcq191 PMC2990663 20880935

[B14] Dilkes B. P. Spielman M. Weizbauer R. Watson B. Burkart-Waco D. Scott R. J. (2008). The maternally expressed WRKY transcription factor TTG2 controls lethality in interploidy crosses of Arabidopsis. PLoS Biol. 6, e308–e2720. 10.1371/journal.pbio.0060308 PMC2596861 19071961

[B15] Doebley J. F. Gaut B. S. Smith B. D. (2006). The molecular genetics of crop domestication. Cell 127, 1309–1321. 10.1016/j.cell.2006.12.006 17190597

[B16] Fossi M. Amundson K. Kuppu S. Britt A. Comai L. (2019). Regeneration of Solanum tuberosum plants from protoplasts induces widespread genome instability. Plant Physiol. 180, 78–86. 10.1104/pp.18.00906 PMC6501065 30792232

[B17] Gahlaut V. Zinta G. Jaiswal V. Kumar S. (2020). Quantitative epigenetics: A new avenue for crop improvement. Epigenomes 4, 25. 10.3390/epigenomes4040025 PMC8594725 34968304

[B18] Gallusci P. Agius D. R. Moschou P. N. Dobránszki J. Kaiserli E. Martinelli F. (2023). Deep inside the epigenetic memories of stressed plants. Trends Plant Sci. 28, 142–153. 10.1016/j.tplants.2022.09.004 36404175

[B19] Gallusci P. Hodgman C. Teyssier E. Seymour G. B. (2016). DNA methylation and chromatin regulation during fleshy fruit development and ripening. Front. Plant Sci. 7, 807. 10.3389/fpls.2016.00807 PMC4905957 27379113

[B20] Gehring M. (2013). Genomic imprinting: Insights from plants. Annu. Rev. Genet. 47, 187–208. 10.1146/annurev-genet-110711-155527 24016190

[B21] Hirochika H. (1993). Activation of tobacco retrotransposons during tissue culture. EMBO J. 12, 2521–2528. 10.1002/j.1460-2075.1993.tb05907.x PMC413490 8389699

[B22] Hollingsworth M. L. Bailey J. P. (2000). Evidence for massive clonal growth in the invasive weed Fallopia japonica (Japanese Knotweed). Botanical J. Linn. Soc. 133, 463–472. 10.1006/bojl.2000.0359

[B23] Huang H. Liu R. Niu Q. Tang K. Zhang B. Zhang H. (2019). Global increase in DNA methylation during orange fruit development and ripening. Proc. Natl. Acad. Sci. U. S. A. 116, 1430–1436. 10.1073/pnas.1815441116 PMC6347674 30635417

[B24] Jablonka E. Lamb M. J. (1998). Epigenetic inheritance in evolution. J. Evol. Biol. 11, 159–183. 10.1046/j.1420-9101.1998.11020159.x

[B25] Kaeppler S. M. Kaeppler H. F. Rhee Y. (2000). Epigenetic aspects of somaclonal variation in plants. Plant Mol. Biol. 43, 179–188. 10.1023/a:1006423110134 10999403

[B26] Kalisz S. Purugganan M. D. (2004). Epialleles via DNA methylation: Consequences for plant evolution. Trends Ecol. Evol. 19, 309–314. 10.1016/j.tree.2004.03.034 16701276

[B27] Köhler C. Dziasek K. Del Toro-De León G. (2021). Postzygotic reproductive isolation established in the endosperm: Mechanisms, drivers and relevance. Philosophical Trans. R. Soc. B Biol. Sci. 376, 20200118. 10.1098/rstb.2020.0118 PMC8101696 33866810

[B28] Liu R. Halle S. Liu M. Kong J. Wu C. Degraeve-Guibault C. (2015). A DEMETER-like DNA demethylase governs tomato fruit ripening. Proc. Natl. Acad. Sci. U. S. A. 112, 10804–10809. 10.1073/pnas.1503362112 PMC4553810 26261318

[B29] Manning K. Tör M. Poole M. Hong Y. Thompson A. J. King G. J. (2006). A naturally occurring epigenetic mutation in a gene encoding an SBP-box transcription factor inhibits tomato fruit ripening. Nat. Genet. 38, 948–952. 10.1038/ng1841 16832354

[B30] Martinez G. Köhler C. (2017). Role of small RNAs in epigenetic reprogramming during plant sexual reproduction. Curr. Opin. Plant Biol. 36, 22–28. 10.1016/j.pbi.2016.12.006 28088028

[B31] McClintock B. (1984). The significance of responses of the genome to challenge. Science 226, 792–801. 10.1126/science.15739260 15739260

[B32] Melquist S. Luff B. Bender J. (1999). Arabidopsis PAI gene arrangements, cytosine methylation and expression. Genetics 153, 401–413. 10.1093/genetics/153.1.401 PMC1460723 10471722

[B33] Mérai Z. Chumak N. García-Aguilar M. Hsieh T.-F. Nishimura T. Schoft V. K. (2014). The AAA-ATPase molecular chaperone Cdc48/p97 disassembles sumoylated centromeres, decondenses heterochromatin, and activates ribosomal RNA genes. Proc. Natl. Acad. Sci. U. S. A. 111, 16166–16171. 10.1073/pnas.1418564111 PMC4234600 25344531

[B34] Mittelsten Scheid O. Afsar K. Paszkowski J. (2003). Formation of stable epialleles and their paramutation-like interaction in tetraploid *Arabidopsis thaliana* . Nat. Genet. 34, 450–454. 10.1038/ng1210 12847525

[B35] Moreno-Romero J. Del Toro-De León G. Yadav V. K. Santos-González J. Köhler C. (2019). Epigenetic signatures associated with imprinted paternally expressed genes in the Arabidopsis endosperm. Genome Biol. 20, 41. 10.1186/s13059-019-1652-0 PMC6385439 30791924

[B36] Naito K. Zhang F. Tsukiyama T. Saito H. Hancock C. N. Richardson A. O. (2009). Unexpected consequences of a sudden and massive transposon amplification on rice gene expression. Nature 461, 1130–1134. 10.1038/nature08479 19847266

[B37] Ong-Abdullah M. Ordway J. M. Jiang N. Ooi S.-E. Kok S.-Y. Sarpan N. (2015). Loss of Karma transposon methylation underlies the mantled somaclonal variant of oil palm. Nature 525, 533–537. 10.1038/nature15365 PMC4857894 26352475

[B38] Phillips R. L. Kaeppler S. M. Olhoft P. (1994). Genetic instability of plant tissue cultures: Breakdown of normal controls. Proc. Natl. Acad. Sci. U. S. A. 91, 5222–5226. 10.1073/pnas.91.12.5222 PMC43966 8202472

[B39] Pouteau S. Grandbastien M.-A. Boccara M. (1994). Microbial elicitors of plant defence responses activate transcription of a retrotransposon. Plant J. 5, 535–542. 10.1046/j.1365-313x.1994.5040535.x

[B40] Pouteau S. Huttner E. Grandbastien M. A. Caboche M. (1991). Specific expression of the tobacco Tnt1 retrotransposon in protoplasts. EMBO J. 10, 1911–1918. 10.1002/j.1460-2075.1991.tb07717.x PMC452866 1710981

[B41] Quadrana L. Almeida J. Asís R. Duffy T. Dominguez P. G. Bermúdez L. (2014). Natural occurring epialleles determine vitamin E accumulation in tomato fruits. Nat. Commun. 5, 4027. 10.1038/ncomms5027 24967512

[B42] Rasmusson D. C. Phillips R. L. (1997). Plant breeding progress and genetic diversity from de novo variation and elevated epistasis. Crop Sci. 37, 303–310. 10.2135/cropsci1997.0011183X003700020001x

[B43] Rokney A. Kobiler O. Amir A. Court D. L. Stavans J. Adhya S. (2008). Host responses influence on the induction of lambda prophage. Mol. Microbiol. 68, 29–36. 10.1111/j.1365-2958.2008.06119.x PMC2327240 18298445

[B44] Saze H. Kakutani T. (2007). Heritable epigenetic mutation of a transposon-flanked Arabidopsis gene due to lack of the chromatin-remodeling factor DDM1. EMBO J. 26, 3641–3652. 10.1038/sj.emboj.7601788 PMC1949009 17627280

[B45] Slotkin R. K. Vaughn M. Borges F. Tanurdzic M. Becker J. D. Feijo J. A. (2009). Epigenetic reprogramming and small RNA silencing of transposable elements in pollen. Cell 136, 461–472. 10.1016/j.cell.2008.12.038 PMC2661848 19203581

[B46] Springer N. M. Schmitz R. J. (2017). Exploiting induced and natural epigenetic variation for crop improvement. Nat. Rev. Genet. 18, 563–575. 10.1038/nrg.2017.45 28669983

[B47] Tsaftaris A. S. Polidoros A. N. (1999). DNA methylation and plant breeding. Plant Breed. Rev. 10.3389/fpls.2021.596236

[B48] Waadt R. Seller C. A. Hsu P.-K. Takahashi Y. Munemasa S. Schroeder J. I. (2022). Plant hormone regulation of abiotic stress responses. Nat. Rev. Mol. Cell Biol. 23, 680–694. 10.1038/s41580-022-00479-6 PMC9592120 35513717

[B49] Weigel D. Colot V. (2012). Epialleles in plant evolution. Genome Biol. 13, 249. 10.1186/gb-2012-13-10-249 PMC3491404 23058244

[B50] Wessler S. R. (1996). Plant retrotransposons: Turned on by stress. Curr. Biol. 6, 959–961. 10.1016/S0960-9822(02)00638-3 8805314

[B51] Whittaker C. Dean C. (2017). The FLC locus: A platform for discoveries in epigenetics and adaptation. Annu. Rev. Cell Dev. Biol. 33, 555–575. 10.1146/annurev-cellbio-100616-060546 28693387

[B52] Yang X. Kundariya H. Xu Y.-Z. Sandhu A. Yu J. Hutton S. F. (2015). MutS HOMOLOG1-derived epigenetic breeding potential in tomato. Plant Physiol. 168, 222–232. 10.1104/pp.15.00075 PMC4424023 25736208

[B53] Yi H. Richards E. J. (2008). Phenotypic instability of Arabidopsis alleles affecting a disease Resistance gene cluster. BMC Plant Biol. 8, 36. 10.1186/1471-2229-8-36 PMC2374787 18410684

[B54] Zeng F. Cheng B. (2014). Transposable element insertion and epigenetic modification cause the multiallelic variation in the expression of FAE1 in sinapis alba. Plant Cell 26, 2648–2659. 10.1105/tpc.114.126631 PMC4114957 24934174

